# Supplementary material for: Seasonal and geographic variation in insecticide resistance in Aedes aegypti in southern Ecuador
Source: PLoS Negl Trop Dis. 2019 Jun 10;13(6):e0007448. doi: 10.1371/journal.pntd.0007448 (PMC6586360; doi:10.1371/journal.pntd.0007448)
Supplement: S1 Table — Significant differences are denoted with an asterisk. (DOCX) [file pntd.0007448.s001.docx]

S1 Table: Post-hoc Fisher’s exact test p-values for Deltamethrin resistance between cities. Significant difference are denoted with an asterisk.

| Season | City | Huaquillas | Machala | Portovelo |
| --- | --- | --- | --- | --- |
| 1 | Machala | < 0.001* |  |  |
|  | Portovelo | 0.002* | 1.00 |  |
|  | Zaruma | 0.008* | < 0.001* | 0.75 |
| 2 | Machala | 0.006* |  |  |
|  | Portovelo | < 0.001* | 1.00 |  |
| 3 | Machala | < 0.001* |  |  |
|  | Portovelo | < 0.001* | < 0.001* |  |
