# Supplementary material for: Seasonal and geographic variation in insecticide resistance in Aedes aegypti in southern Ecuador
Source: PLoS Negl Trop Dis. 2019 Jun 10;13(6):e0007448. doi: 10.1371/journal.pntd.0007448 (PMC6586360; doi:10.1371/journal.pntd.0007448)
Supplement: S2 Table — Significant differences are denoted with an asterisk. (DOCX) [file pntd.0007448.s002.docx]

S2 Table: Post-hoc Fisher’s exact test *p*-values for Alpha-Cypermethrin resistance between cities in the season two collection period. Significant difference are denoted with an asterisk.

| City | Huaquillas | Machala |
| --- | --- | --- |
| Machala | <0.001* |  |
| Portovelo | 0.70 | <0.001* |
