# Supplementary material for: Seasonal and geographic variation in insecticide resistance in Aedes aegypti in southern Ecuador
Source: PLoS Negl Trop Dis. 2019 Jun 10;13(6):e0007448. doi: 10.1371/journal.pntd.0007448 (PMC6586360; doi:10.1371/journal.pntd.0007448)
Supplement: S4 Table — Significant differences are denoted with an asterisk. (DOCX) [file pntd.0007448.s004.docx]

S4 Table: Post-hoc Fisher’s exact test *p*-values for Alpha-cypermethrin resistance between seasons. Significant difference are denoted with an asterisk.

| **City** | **Season** | **1** | **2** |
| --- | --- | --- | --- |
| Machala | 2 | <0.001* |  |
|  | 3 | <0.001* | 0.05* |
| Portovelo | 3 |  | 0.24 |
