# Supplementary material for: Seasonal and geographic variation in insecticide resistance in Aedes aegypti in southern Ecuador
Source: PLoS Negl Trop Dis. 2019 Jun 10;13(6):e0007448. doi: 10.1371/journal.pntd.0007448 (PMC6586360; doi:10.1371/journal.pntd.0007448)
Supplement: S6 Table — Significant differences are denoted with an asterisk. (DOCX) [file pntd.0007448.s006.docx]

S6 Table: Post-hoc Fisher’s exact test *p*-values for genotype V1016I in season 1, with comparisons in genotype frequencies made between the cities of Huaquillas (H), Machala (M), Portovelo (P), and Zaruma (Z). Significant difference are denoted with an asterisk.

| City:City^a^ | I/I : V/I | I/I : V/V | V/I : V/V |
| --- | --- | --- | --- |
| H:M | < 0.001* | 1.00 | 1.00 |
| H:P | 0.03* | 0.94 | 1.00 |
| H:Z | 0.44 | 1.00 | 0.32 |
| M:P | 1.00 | 1.00 | 1.00 |
| M:Z | 0.09 | 0.08 | 1.00 |
| P:Z | 1.00 | 0.05* | 1.00 |

^a^H – Huaquillas, M-Machala, P-Portovelo, Z-Zaruma
