# Supplementary material for: Seasonal and geographic variation in insecticide resistance in Aedes aegypti in southern Ecuador
Source: PLoS Negl Trop Dis. 2019 Jun 10;13(6):e0007448. doi: 10.1371/journal.pntd.0007448 (PMC6586360; doi:10.1371/journal.pntd.0007448)
Supplement: S8 Table — Significant differences are denoted with an asterisk. (DOCX) [file pntd.0007448.s008.docx]

S8 Table: Post-hoc Fisher’s exact test *p*-values for genotype V1016I in Machala, with comparisons in genotype frequencies made between seasons. Significant difference are denoted with an asterisk.

| Season:  Season | I/I :  V/I | I/I : V/V | V/I : V/V |
| --- | --- | --- | --- |
| 1:2 | 1.00 | 1.00 | 1.00 |
| 1:3 | 1.00 | 0.02 | 1.00 |
| 2:3 | 0.02* | 0.43 | 1.00 |
