# Supplementary material for: Seasonal and geographic variation in insecticide resistance in Aedes aegypti in southern Ecuador
Source: PLoS Negl Trop Dis. 2019 Jun 10;13(6):e0007448. doi: 10.1371/journal.pntd.0007448 (PMC6586360; doi:10.1371/journal.pntd.0007448)
Supplement: S9 Table — Significant differences are denoted with an asterisk. (DOCX) [file pntd.0007448.s009.docx]

S9 Table. Post-hoc Fisher’s exact test *p*-values for genotype V1016I in Portovelo, with comparisons in genotype frequencies made between seasons. Significant difference are denoted with an asterisk.

| Season:  Season | I/I :  V/I | I/I : V/V | V/I : V/V |
| --- | --- | --- | --- |
| 1:2 | 0.05* | 0.36 | 1.00 |
| 1:3 | 0.001* | 0.005* | 1.00 |
| 2:3 | 1.00 | 0.58 | 1.00 |
