# Supplementary material for: Seasonal and geographic variation in insecticide resistance in Aedes aegypti in southern Ecuador
Source: PLoS Negl Trop Dis. 2019 Jun 10;13(6):e0007448. doi: 10.1371/journal.pntd.0007448 (PMC6586360; doi:10.1371/journal.pntd.0007448)
Supplement: S11 Table — (DOCX) [file pntd.0007448.s011.docx]

S11 Table. V1016I and F1534C Genotypes compared to alpha-cypermethrin resistant phenotype

| V1016I |  | I/I  (mutant) | V/I  (heterozygote) | V/V  (wild type) | *p*-value^a^ |
| --- | --- | --- | --- | --- | --- |
|  | *Resistant* | 80 | 58 | 2 | <0.001* |
|  | *Susceptible* | 23 | 84 | 32 |  |
| F1534C |  | C/C  (mutant) | F/C  (heterozygote) | F/F  (wild type) |  |
|  | *Resistant* | 138 | 2 | 0 | 0.10 |
|  | *Susceptible* | 132 | 7 | 0 |  |

^a^Fisher’s Exact Test
