# Supplementary material for: Seasonal and geographic variation in insecticide resistance in Aedes aegypti in southern Ecuador
Source: PLoS Negl Trop Dis. 2019 Jun 10;13(6):e0007448. doi: 10.1371/journal.pntd.0007448 (PMC6586360; doi:10.1371/journal.pntd.0007448)
Supplement: S12 Table — Significant p-values indicating the population is likely not in Hardy Weinberg equilibrium are denoted with an asterisk. (DOCX) [file pntd.0007448.s012.docx]

S12 Table. Exact test *p*-values for the exact test of Hardy-Weinberg equilibrium for the V1016I genotype frequencies in each city and season. Significant *p*-values indicating the population is likely not in Hardy Weinberg equilibrium are denoted with an asterisk.

|  | **Season** | | |
| --- | --- | --- | --- |
| **City** | **1** | **2** | **3** |
| Huaquillas | 0.03* | 0.10 | 0.73 |
| Portovelo | 1.00 | 0.06 | 0.02* |
| Machala | 0.03* | 0.63 | 0.18 |
| Zaruma | 0.11 | na | na |
