# Supplementary figures and images for: Seasonal and geographic variation in insecticide resistance in Aedes aegypti in southern Ecuador
Source: PLoS Negl Trop Dis. 2019 Jun 10;13(6):e0007448. doi: 10.1371/journal.pntd.0007448 (PMC6586360; doi:10.1371/journal.pntd.0007448)

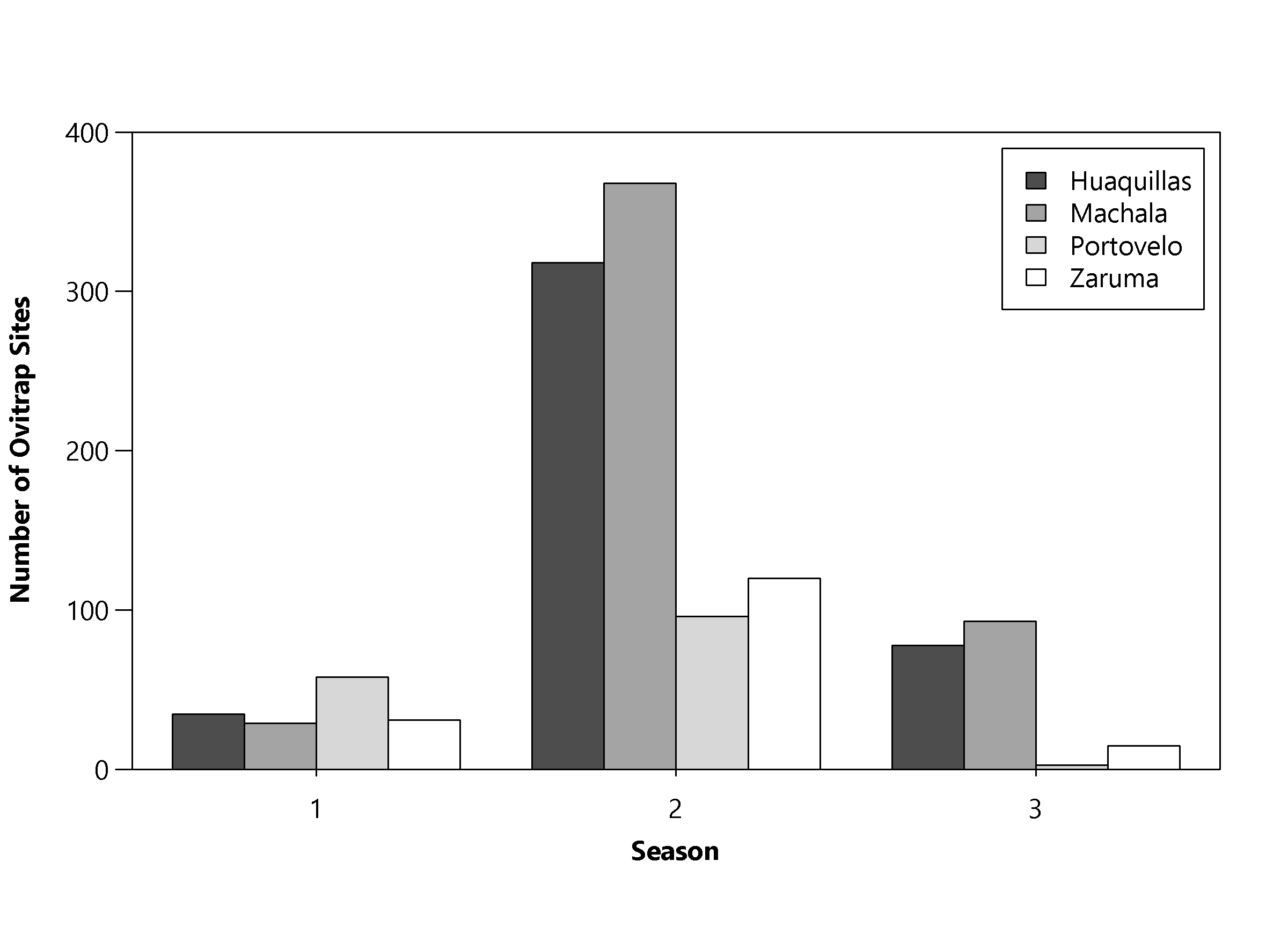

Supplement: S1 Fig — Total weekly dengue cases reported to the Ecuador Ministry of Health are given for Huaquillas, Machala, Portovelo, and Zaruma (TIFF) [file pntd.0007448.s013.tiff]

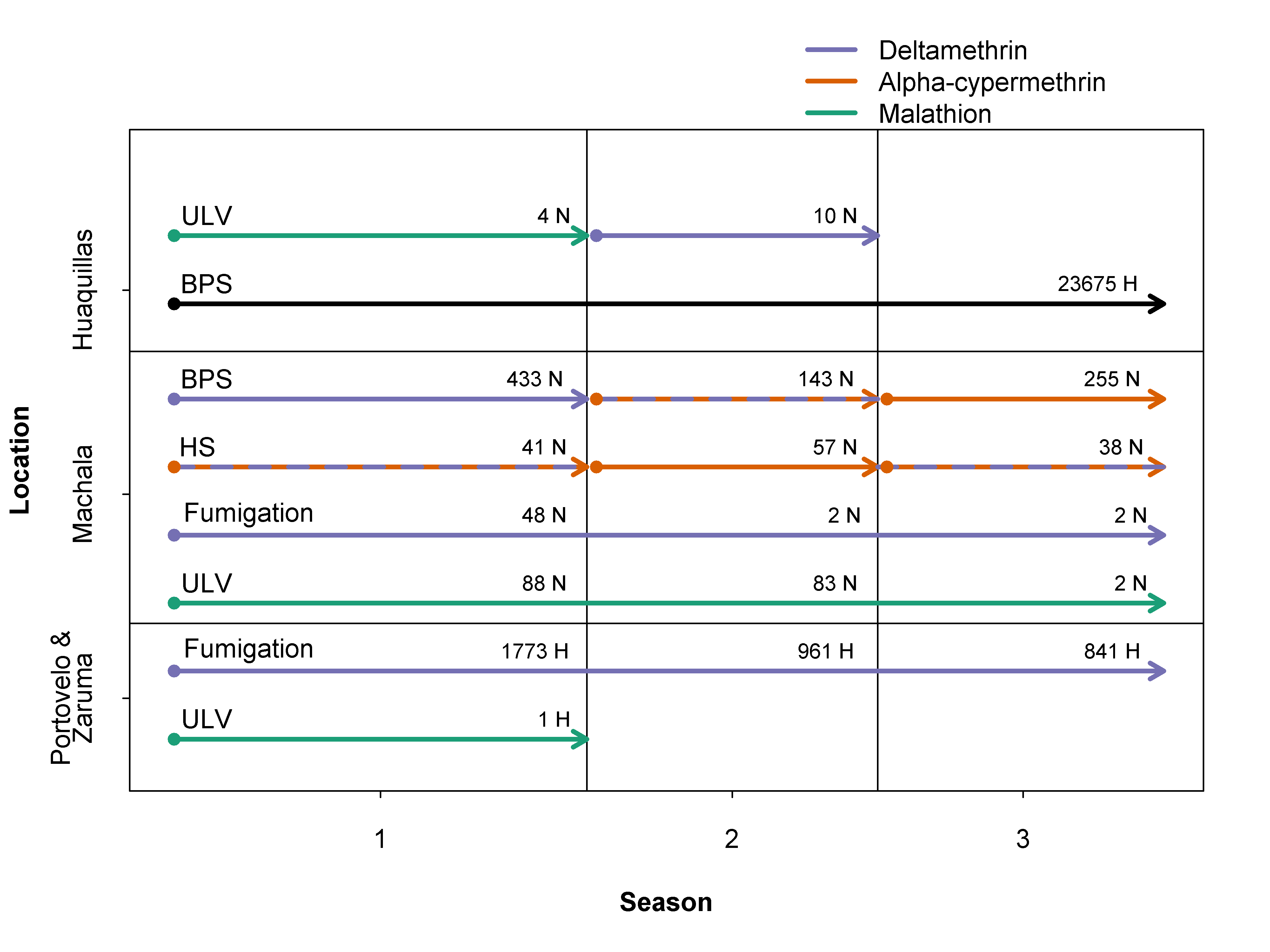

Supplement: S2 Fig — (TIFF) [file pntd.0007448.s014.tiff]

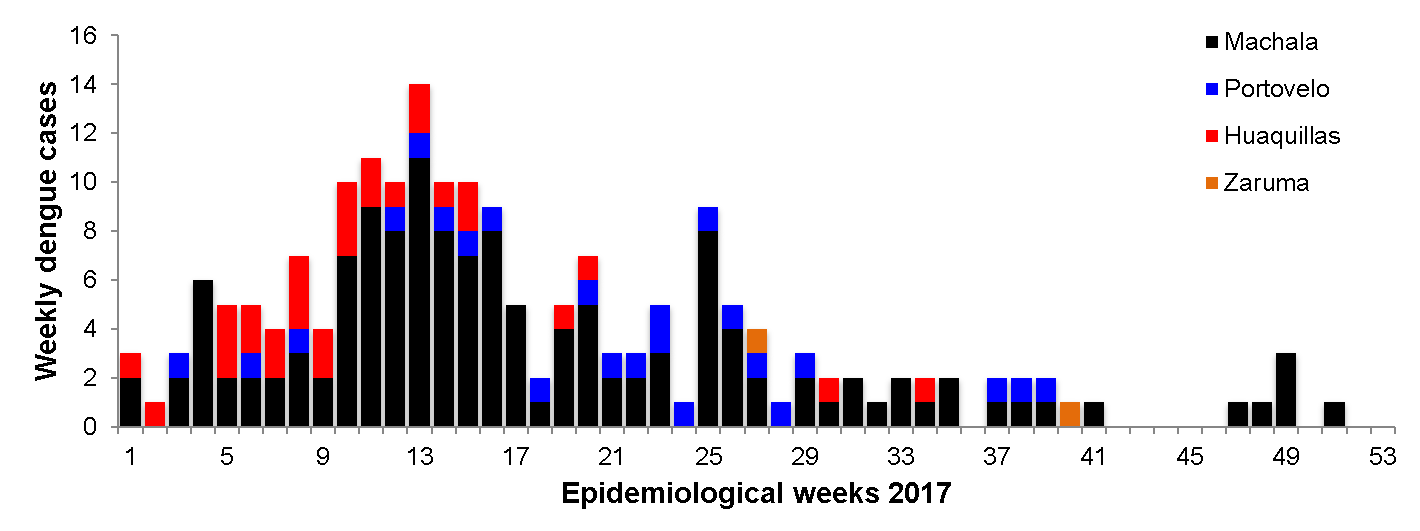

Supplement: S3 Fig — Dissociation curves representing the susceptible (wild type) homozygous (A), heterozygous (B), and resistant (mutant) heterozygous (C) genotypes. (TIF) [file pntd.0007448.s015.tif]

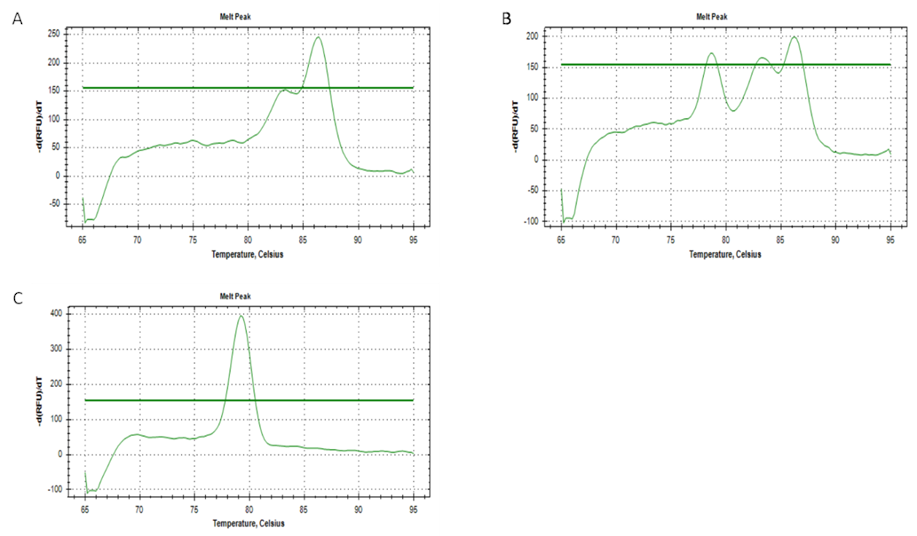

Supplement: S4 Fig — Dissociation curve representing the wild type (susceptible) homozygous (A), heterozygous (B), and mutant (C) genotypes. (TIF) [file pntd.0007448.s016.tif]

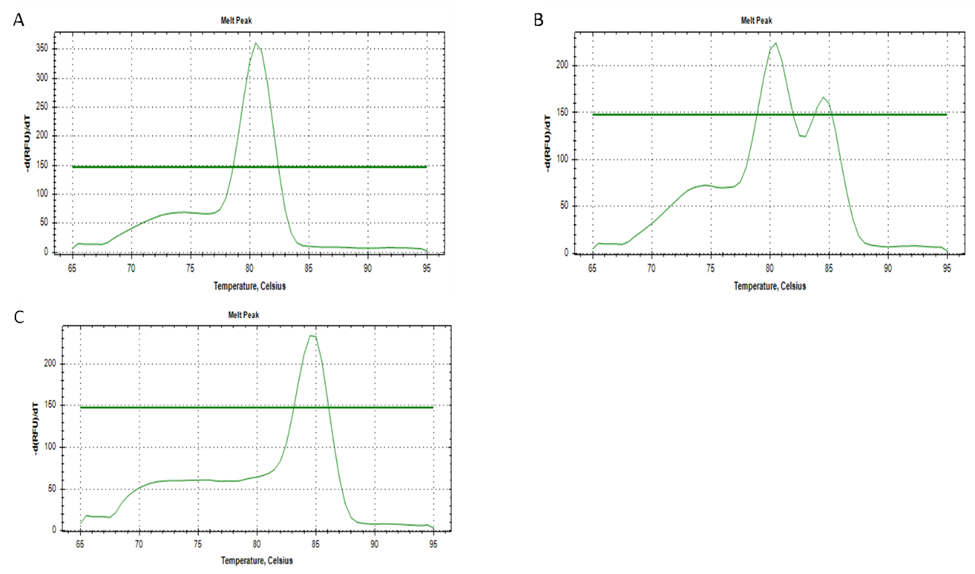

Supplement: S5 Fig — Timing, application method, and number of houses or neighborhoods treated are shown. Dashed arrows represent a mixture of two insecticides. One insecticide product (black) was unidentified. The house number for Huaquillas represents total houses treated over the season. ULV = ultra low-volume spraying, BPS = backpack spraying, HS = handpump spraying, H = houses, N = neighborhoods. (TIF) [file pntd.0007448.s017.tif]
